# The Role of Nutrition in Obesity

**DOI:** 10.3390/nu15112556

**Published:** 2023-05-30

**Authors:** Jingjing Jiao

**Affiliations:** Department of Nutrition, School of Public Health & Department of Endocrinology, The Second Affiliated Hospital, Zhejiang University School of Medicine, Hangzhou 310058, China; jingjingjiao@zju.edu.cn

This Special Issue of *Nutrients*, “Nutrients, Foods, Dietary Patterns and Obesity”, includes nine original articles that explore the role of eating behaviors, diet quality and dietary interventions in obesity and its potential mechanisms. It also contains one systematic review and meta-analysis investigating the effectiveness of very-low-calorie diets and low-fat vegan diets on weight and glycemic markers in diabetic patients.

The prevalence of obesity has been increasing in both developed and low- and middle-income countries, accompanied by increased incidence of obesity-related chronic diseases. Nevertheless, the health consequences of obesity are often disregarded in Sub-Saharan Africa. Ruben and colleagues quantified the overweight and obesity burden among adults in urban Bissau [1], which is similar to the trends observed in many other urbanized populations in Africa and highlights the need for policies and strategies to curb this public health issue.

The main cause of obesity is thought to be overeating, which leads to chronic positive energy balance. Haofeng and colleagues examined the psychometric properties of the Addiction-like Eating Behavior Scale (AEBS) among the Chinese population and found positive associations with modern eating-related habits, including ordering delivery food, eating late-night meals and watching mukbang videos [2]. Another study in this Special Issue shows that boredom was most strongly associated with the urge to eat [3]. Compared to men, women seemed to be more inclined towards emotional eating, especially in response to feelings such as depression and anger [3]. These findings may help researchers develop personalized strategies for the control of unhealthy eating habits to treat obesity.

In addition to quantity, diet quality also determines the obesogenic effects of foods and affects metabolic health through diverse biological pathways. Based on EAT-Lancet recommendations, Fabricio and colleagues developed a sustainable dietary score (SDS) that incorporated characteristics of the Mexican population [4]. They used the SDS to evaluate the diet quality of the Mexican population; this tool may also be useful for monitoring the progress of a dietary intervention. Interestingly, one study in this Special Issue suggests a negative interaction between diet quality and heavy metals in relation to the development of obesity [5]. Using the data from the National Health and Examination Survey (2007–2018), Tiezheng and colleagues reported that the inverse association of the HEI-2015 scores with peripheral obesity was attenuated by higher levels of heavy metals. Thus, dietary interventions for the purpose of weight loss may take heavy metals into consideration, which could counteract the beneficial effect of a healthy diet.

Previous evidence has suggested that consumption of low-energy-dense (LED) foods, such as fish, lean meat, fruits and vegetables, could reduce hunger sensations and energy intake and thus help with weight loss. Sophie and colleagues conducted a randomized crossover study to compare the influence of low energy-dense ready meals (LEDRMs) with higher energy-dense ready meals (HEDRM) on satiety and food intake [6]. Although LEDRM did not lead to a lower energy intake, it increased fullness and could help improve the nutritional content of meals, decreasing the intake of saturated fat [6]. Similarly, whole-grain-containing diets prevent obesity by modulating metabolic functions and pro-inflammatory states. Another intervention study by Wei-Yi and colleagues evaluated the effects of Dehulled Adlay consumption on lipid metabolism and inflammation in overweight and obese individuals [7]. After a 6-week intervention, they found taking 60 g of dehulled adlay powder per day significantly improved body fat mass, blood levels of total cholesterol, triglyceride and inflammatory indicators [7]. Weight loss is also protective for clinical outcomes among patients with type 2 diabetes (T2D). To evaluate the role of calorie restriction and vegan diets in weight and glycemic control in T2D patients, Anjali and colleagues summarized evidence from 16 clinical trials (*n* = 834) using very low-calorie diets (VLCD) or vegan diets [8]. They showed that the LDL cholesterol level was significantly decreased by the vegan diet, while VLCD improved glycaemia control in T2D patients. A trend toward decreased body weight was observed for both diets. These findings may be useful for formulating national guidelines and recommendations to manage obesity and diabetes.

Maternal overweight and obesity has been linked with an increased risk of developing chronic diseases in offspring. However, the underlying mechanisms remain unclear. In a cross-sectional study by Martha and colleagues, the abundance of Bacteroidetes and Actinobacteria in human milk was directly and significantly associated with the adiposity of the woman before pregnancy and during lactation [9], suggesting intestinal microbiota play an important role in mediating the impact of nutrition status of mothers on their offspring. In terms of the precise mechanism, one experimental study in this Special Issue revealed that the complex of phycobiliproteins, fucoxanthin, and krill oil reduced body weight and improved blood lipid profile in rats fed a high-fat diet, inhibiting the enzyme activities of lipid synthesis and enhancing antioxidant activity [10].

In conclusion, eating behaviors, diet quantity and quality, as well as other environmental factors, such as heavy metals, should all be considered in the development of strategies for obesity prevention and management. A vegan diet and VLCD could be effective for improving blood LDL cholesterol and glucose control, respectively, among T2D patients. Gut microbiota may be the mediator for the effect of maternal obesity on offspring. More studies are needed to unravel the precise mechanisms of individual nutrients, foods or dietary patterns in regulating lipid metabolism and thus promote the development of personalized dietary treatment for obesity.

## References

[1] Turé R., Damasceno A., Djicó M., Lunet N. (2021). Prevalence of Underweight, Overweight and Obesity among Adults in Urban Bissau, Western Africa. Nutrients.

[2] Ling H., Chen J.H., Tong K.K., Dang L., Wu A.M.S. (2022). Addiction-like Eating in Chinese Adults: An Assessment Tool and Its Associations with Modern Eating-Related Habits. Nutrients.

[3] Guerrero-Hreins E., Stammers L., Wong L., Brown R.M., Sumithran P. (2022). A Comparison of Emotional Triggers for Eating in Men and Women with Obesity. Nutrients.

[4] Campirano F., López-Olmedo N., Ramírez-Palacios P., Salmerón J. (2023). Sustainable Dietary Score: Methodology for Its Assessment in Mexico Based on EAT-Lancet Recommendations. Nutrients.

[5] Li T., Yu L., Yang Z., Shen P., Lin H., Shui L., Tang M., Jin M., Chen K., Wang J. (2022). Associations of Diet Quality and Heavy Metals with Obesity in Adults: A Cross-Sectional Study from National Health and Nutrition Examination Survey (NHANES). Nutrients.

[6] Hannon S.C., Hillier S.E., Thondre P.S., Clegg M.E. (2021). Lower Energy-Dense Ready Meal Consumption Affects Self-Reported Appetite Ratings with No Effect on Subsequent Food Intake in Women. Nutrients.

[7] Cheng W.-Y., Yeh W.-J., Ko J., Huang Y.-L., Yang H.-Y. (2022). Consumption of Dehulled Adlay Improved Lipid Metabolism and Inflammation in Overweight and Obese Individuals after a 6-Week Single-Arm Pilot Study. Nutrients.

[8] Kashyap A., Mackay A., Carter B., Fyfe C.L., Johnstone A.M., Myint P.K. (2022). Investigating the Effectiveness of Very Low-Calorie Diets and Low-Fat Vegan Diets on Weight and Glycemic Markers in Type 2 Diabetes Mellitus: A Systematic Review and Meta-Analysis. Nutrients.

[9] Chavoya-Guardado M.A., Vasquez-Garibay E.M., Ruiz-Quezada S.L., Ramírez-Cordero M.I., Larrosa-Haro A., Castro-Albarran J. (2022). *Firmicutes*, *Bacteroidetes* and *Actinobacteria* in Human Milk and Maternal Adiposity. Nutrients.

[10] Qiang X., Guo C., Gu W., Song Y., Zhang Y., Gong X., Wang L., Wang G. (2022). The Complex of Phycobiliproteins, Fucoxanthin, and Krill Oil Ameliorates Obesity through Modulation of Lipid Metabolism and Antioxidants in Obese Rats. Nutrients.

